# Thoracic perfusion of recombinant mutant human tumor necrosis factor (rmhTNF) can be considered as a good adjunct in the treatment of malignant pleural effusion caused by lung cancer

**DOI:** 10.1186/s12890-020-01210-x

**Published:** 2020-06-18

**Authors:** Tian Fu, Yong Lin, Qingdi Zeng, Wei Yao, Liping Han

**Affiliations:** 1 Department of Respiration, Jining NO.1 People’s Hospital, Jining, 272011 Shandong Province China; 2 Department of Clinical Laboratory, Jining NO.1 People’s Hospital, Jining, 272011 Shandong Province China; 3 General surgery, Zoucheng Kanzhuang Township Health Center, Zoucheng, 273502 Shandong Province China

**Keywords:** Recombinant mutant human tumor necrosis factor, RmhTNF, Malignant pleural effusion, Lung cancer, Meta-analysis

## Abstract

**Background:**

Tumor necrosis factor (TNF) has been investigated to be correlated with the occurrence and progression of lung cancer. This investigation was to assess the efficacy and safety of recombinant mutant human tumor necrosis factor (rmhTNF) for controlling malignant pleural effusion (MPE) through thoracic perfusion.

**Methods:**

Through searching from MEDLINE, Web of Science, EMBASE, Cochrance Library, OVID and China National Knowledge Infrastructure (CNKI), a total of 12 studies with 694 patients were included in this study. A series of meta-analysis methods were used to analyze the extracted data.

**Results:**

Thoracic perfusion of rmhTNF combined with cisplatin promoted the objective response rate (ORR) (P < 0.001; odds ratio = 4.49) and the quality of life (QOL) of patients with MPE (P < 0.001; odds ratio = 10.33), as compared with cisplatin alone. Although the participation of rmhTNF increased the incidence of fever (P < 0.001), it seemed to relieve the adverse reactions in the digestive tract (P = 0.017).

**Conclusions:**

Thoracic perfusion of rmhTNF contributes to the treatment of MPE and improves the QOL of MPE patients.

## Background

Malignant pleural effusion (MPE) refers to the presence of neoplastic cells in the pleural fluid, which may involve in the complex interaction between pleural mesothelial cells and malignant tumor cells [1]. Statistical data suggest that lung cancer, breast cancer and lymphoma are the most common causes of MPE, especially lung cancer [2]. The MPE is closely related to the survival of lung cancer patients, which cuts down the quality of life (QOL) by causing dyspnea and chest pain [3]. Currently, some chemotherapy drugs, immune and biological agents have been tried to inject into the pleural cavity for controlling the progress of MPE [4–6]. In 1975, a cytotoxic factor produced by macrophages is reported and named as tumor necrosis factor (TNF) [7]. TNF is a multifunctional cytokine that can directly kill or inhibit tumor cells and so play an important role in tumorigenesis and development [7, 8]. The human TNF gene maps to chromosome 6p21.3, spans about 3 kilobases and contains 4 exons [9], which produced a 233-amino acid-long type II transmembrane protein arranged in stable homotrimers [10]. The primary role of TNF is in the regulation of immune cells and it is able to induce apoptosis, inhibit tumorigenesis [11] and retard the proliferation, angiogenesis and metastasis of cancer cells [12].

In China, a new recombinant mutant human tumor necrosis factor (rmhTNF) has been developed, which is a product obtained by modifying the TNF gene using the polymerase chain reaction technology based on the tumor necrosis factor cDNA template prototype, resulting in a non-glycosylated single chain consisting of 151 amino acids with a molecular weight of 16,598 Da [13]. The feature of rmhTNF is the deletion of the first seven amino acids and substitution of four amino acids (Arg for Pro at position 8, Lys for Ser at position 9, Arg for Asp at position 10, and Phe for Leu at position 157) and thus enhances the anti-tumor activity [14]. In 2004, the rmhTNF is approved by the China State Food and Drug Administration (SFDA) for the treatment of cancer. As yet, some studies have reported that the rmhTNF can be used to treat the MPE by thoracic perfusion. This research is a systematic evaluation and meta-analysis to quantify the efficacy and safety of rmhTNF in the treatment of MPE caused by lung cancer.

## Methods

### Searching and identification of studies

From Medline/PubMed, Embase, OVID, SpringerLink, CochraneLibrary, Web of Science and CNKI (Chinese National Knowledge Infrastructure), we searched the studies on the efficacy and safety of rmhTNF in the treatment of MPE through thoracic perfusion (from January 2000 to December 2018) with the key words including “malignant pleural effusion,” “MPE,” “lung cancer,” “recombinant mutant human tumor necrosis factor,” “rmhTNF,” “thoracic perfusion,” “intrathoracic injection,” and “intrapleural injection”. The search strategy was to combine the topic of rmhTNF with the topic of MPE, and to combine them with thoracic perfusion. The study was conducted according to the Preferred Reporting Items for Systematic Reviews and Meta-Analyses (PRISMA) statement [15].

### Inclusion criteria for selecting studies

Inclusion criteria: (1) patients must be diagnosed with lung cancer and malignant tumor cells must be identified from the pleural cavity by cytology and histology; (2) study must compare the efficacy and safety between rmhTNF plus cisplatin and cisplatin alone by thoracic perfusion for treating MPE; (3) the supportive treatment and the clinical baseline of two groups must be basically equal; and (4) the outcome measures, including objective response rate (ORR), disease control rate (DCR), symptom improvement (SI) and adverse effects (AEs) must be reported.

### Exclusion criteria for selecting studies

Exclusion criteria: (1) non-original articles, such as abstract, meeting record, editorial and review; (2) non-human studies; (3) the research funding was provided by the producer of rmhTNF; (4) loss rate of patients was above 15%; and (5) the study quality was low (by the evaluation criteria from the Cochrane Handbook Version 5.0.1); and (6) lack of ethics statement.

### Extraction of study variables

The variables we extracted included: (1) authors, publication years, the size of study; (2) gender and histology; (3) the QOL; (4) the specific process of clinical intervention; (5) ORR and DCR; and (6) AEs.

### Screening of clinical intervention

Design ideas: efficacy and safety evaluation of rmhTNF combined with cisplatin versus cisplatin alone by thoracic perfusion in treatment of MPE. Implementation: the dosage of rmhTNF was determined by the instruction of producer and the frequency of dosing was administrated each week (at least two times). Measurements of efficacy: ORR, DCR and QOL. Safety evaluation: AEs [16].

### Efficacy evaluation criteria for treating MPE

All studies must have adopted the criteria recommended by WHO to evaluate the treatment efficacy [17]. Complete response (CR): pleural effusion completely disappeared at least 4 weeks or more; partial response (PR): pleural effusion was significantly reduced (> 50%) and maintained for more than 4 weeks; stable disease (SD): reduced pleural effusion < 50% or increased < 25%; progressive disease (PD): pleural effusion increased by > 25%. The overall response rate (ORR) was defined as CR + PR/overall cases and no response rate (NRR) calculated as SD + PD/overall cases.

### Quality evaluation of included studies

We used the Cochrane manual (version 5.1.0) to assess the clinical and statistical design quality of the study [18]. The criteria include the following aspects: (1) whether to describe random grouping and how to generate a random sequence; (2) blind description; (3) allocation concealment; (4) description of outcome data; (5) reporting of selective outcome; (6) other bias factors; and (7) intention-to-treat (ITT) .

### Statistical ideas

The statistical ideas are as follows: (1) two different statistical models, fixed effect model and random effect model, were used to quantify the data; (2) the chi-square test and I^2^ were used to measure the heterogeneity of the included studies; (3) the statistical effects of observed variables were calculated by odds ratios (OR) and 95% confidence interval (CI); (4) the overall effect of observation indicators were measured by the z-value; (5) a P-value less than 0.05 was considered statistically significant; (6) a sensitivity analysis on the included studies was performed to determine the stability of the overall effect; (7) the funnel plot, Begg’s test and Egger’s test were used to assess the possibility of publication bias; (8) the descriptive statistics of measurement data were analyzed by SPSS software (version 23.0, IBM Corporation); (9) the RevMan 5.2 (Cochrane collaboration) and Stata version 14.0 (Stata Corporation) were employed to perform the meta-analysis.

## Results

### A total of 12 studies are included in this meta-analysis

**Fig. 1:**
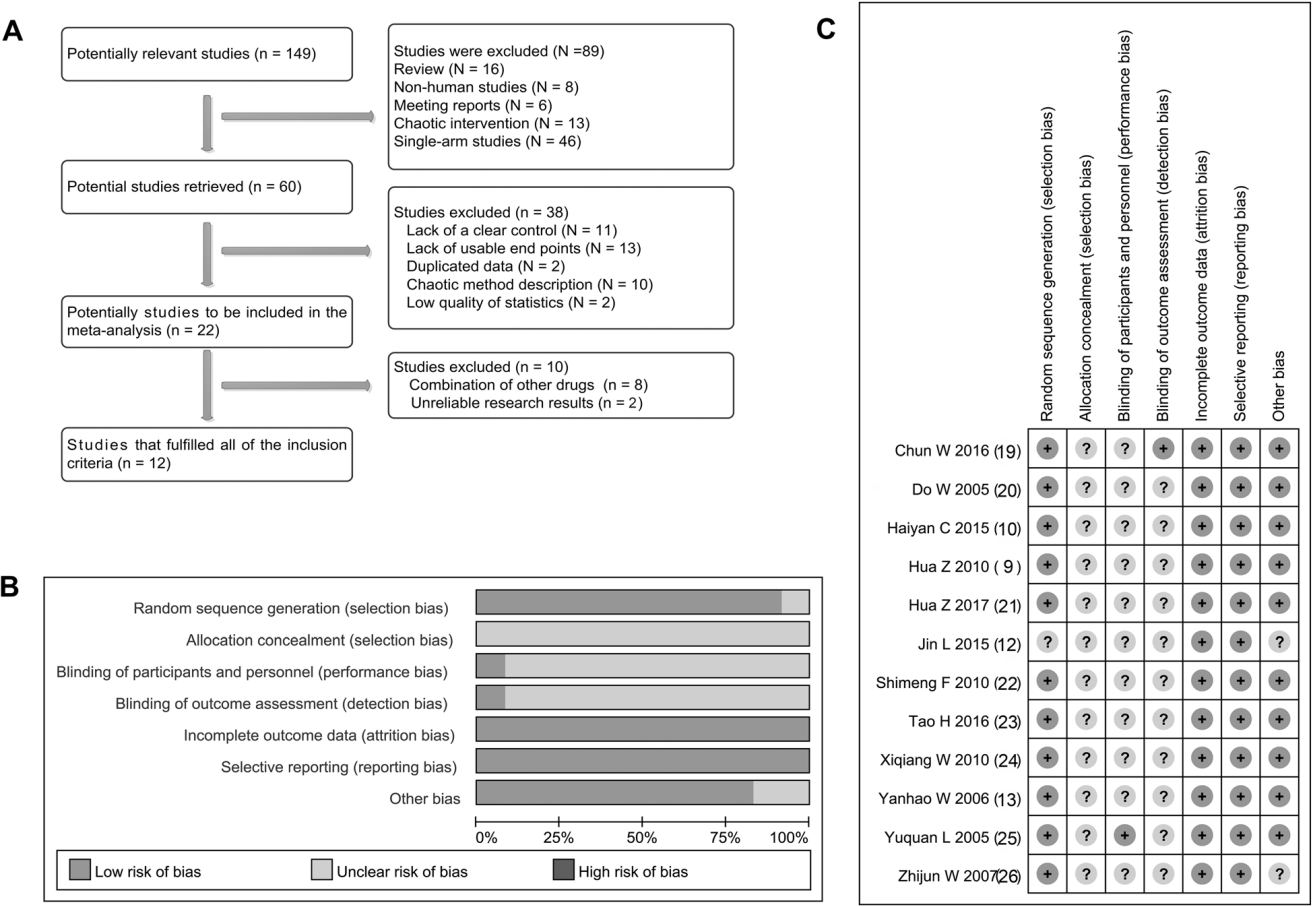
Screening and quality evaluation of included studies. **a** A total of 12 studies are recruited from PubMed, Embase, Cochrane Library, OVID, SCI and CNKI databases. **b**, **c** The quality of the included studies is evaluated using the criteria established by the Cochrane manual (5.0.1 edition), and the results suggests that the included studies are of medium quality

Initially, 149 studies were founded to be closely related to the design of our meta-analysis. However, one hundred and thirty-seven studies were not qualified for inclusion criterion. Finally, a total of 12 studies [9, 10, 12, 13, 19–26] met the inclusion were recruited in this meta-analysis. The detailed search and screening process is shown in Fig. 1a.

### The baseline clinical feature of included studies shows a good consistency

A total of 12 studies [9, 10, 12, 13, 19–26] containing 694 patients were included in this meta-analysis. Of these, 60% were male and 40% were female. The histological types included lung adenocarcinoma (LAC, 75.2%), lung squamous cell carcinoma (LSCC, 18.2%), small cell lung cancer (SCLC, 2.9%), adenosquamous carcinoma (ADSC, 2.7%) and other types (1%). The clinical feature of included studies is shown in detail in Table 1.

**Table 1:** Patient characteristics of the clinical trials

Study	N	Male	Female	Age (Years)	Histology of Lung cancer						Quality of Life	End point
					LAC	LSCC	SCLC	LCLC	ADSC	Other		
Yuquan L 2005 [25]	36	25	11	–	29		7	0	0	0	KPS ≥ 50	ORR, DCR, AEs
Do W 2005 [20]	62	–	–	24–81	–	–	–	–	–	–	KPS ≥ 50	ORR, DCR, AEs
Yanhao W 2006 [13]	56	37	19	24–80	40	5	6	0	3	2	KPS ≥ 50	ORR, DCR, AEs
Zhijun W 2007 [26]	84	61	23	36–73	56	16	0	0	12	0	ECOG≤2	ORR, DCR, AEs
Shimeng F 2010 [22]	73	43	30	38–76	51	11	11	0	0	0	KPS ≥ 60	ORR, DCR, AEs
Hua Z 2010 [9]	58	31	27	39–81	47	10	0	0	1	0	KPS ≥ 60	ORR, DCR, AEs
Xiqiang W 2010 [24]	54	–	–	36–72	32	19	0	3	0	0	ECOG≤2	ORR, DCR, AEs
Haiyan C 2015 [10]	53	25	28	40–81	36	13	−0	0	0	4	KPS ≥ 60	ORR, DCR, AEs
Jin L 2015 [12]	55	–	–	62.5	55	0	0	0	0	0	KPS ≥ 60	ORR, DCR, SI, AEs
Tao H 2016 [23]	60	38	22	41–78	60						–	ORR, DCR, AEs
Chun W 2016 [19]	64	35	29	65–89	37	27	0	0	0	0	–	ORR, DCR, AEs
Hua Z 2017 [21]	39	19	20	61–88	32	7	0	0	0	0	KPS ≥ 50	ORR, DCR, AEs

*N* number of patients, *M/F* male/ female, *LAC* lung adenocarcinoma, *LSCC* lung squamous cell carcinoma, *SCLC* small cell lung cancer, *LCLC* large cell lung cancer, *ADSC* adenosquamous carcinoma, *KPS* Karnofsky performance scale index, *ECOG* performance scale index made by Eastern Cooperative Oncology Group, *ORR* objective response rate, *DCR* disease control rate, *SI* symptom improvement, *AEs* adverse effects

### Quality assessment of included studies

As shown in Table 2, all 12 studies [9, 10, 12, 13, 19–26] were single-center retrospective studies. The eight studies [9, 10, 13, 19–23] were grouped using a random method. However, all studies [9, 10, 12, 13, 19–26] did not perform allocation concealment and only 2 studies [19, 23] provided the information on blind. All studies described outcome measures and did not exist a selective reporting [9, 10, 12, 13, 19–26]. Ten studies [9, 10, 12, 13, 20–22, 24–26] showed unclear bias risk and 2 [19, 23] displayed low risk. Overall, the included studies had a moderate research quality (Fig. 1b and c).

**Table 2:** Raw data and methodological quality of included trials

Studies	Region	Sequence generation	Allocation concealment	Blind	Outcome data	Selective outcome reporting	Other sources of bias	ITT	Risk of bias
Yuquan L 2005 [25]	Single center	–	Insufficient	Unclear	Yes	No	Unclear	Yes	Unclear risk of bias
Do W 2005 [20]	Single center	Random number table (SPSS)	Insufficient	Unclear	Yes	No	Unclear	Yes	Unclear risk of bias
Yanhao W 2006 [13]	Single center	Random number table (SPSS)	Insufficient	Unclear	Yes	No	Unclear	Yes	Unclear risk of bias
Zhijun W [26]	Single center	–	Insufficient	Unclear	Yes	No	Unclear	Yes	Unclear risk of bias
Shimeng F 2010 [22]	Single center	Random number table (SPSS)	Insufficient	Unclear	Yes	No	Unclear	Yes	Unclear risk of bias
Hua Z 2010 [9]	Single center	Random number table (SPSS)	Insufficient	Unclear	Yes	No	Unclear	Yes	Unclear risk of bias
Xiqiang W 2010 [24]	Single center	–	Insufficient	Unclear	Yes	No	Unclear	Yes	Unclear risk of bias
Haiyan C 2015 [10]	Single center	Random number table (SAS)	Insufficient	Unclear	Yes	No	Unclear	Yes	Unclear risk of bias
Jin L 2015 [12]	Single center	–	Insufficient	Unclear	Yes	No	Unclear	Yes	Unclear risk of bias
Tao H 2016 [23]	Single center	Random number table (SPSS)	Insufficient	Clear	Yes	No	Unclear	Yes	Low risk of bias
Chun W 2016 [19]	Single center	Random number table (SPSS)	Insufficient	Clear	Yes	No	Unclear	Yes	Low risk of bias
Hua Z 2017 [21]	Single center	Random number table (SPSS)	Insufficient	Unclear	Yes	No	Unclear	Yes	Unclear risk of bias

*ITT* intention-to-treat

### The included studies display a good comparability

As shown in Table 3, there were 365 patients in the trial group and 329 in the control group. The medication regimen of the observation group was thoracic perfusion combined with rmhTNF and cisplatin. The control group’s regimen was pleural perfusion of cisplatin alone. The dose of rmhTNF was depended on the manufacturer’s instructions and the frequency was one time a week, at least 2 times or until pleural effusion disappeared. Summary analysis suggested that the included studies have a good comparability.

**Table 3:** Assessment of administration of included studies

Study	Trial group (N)	Control Group (N)	Interventions (Groups)		Treatment cycle	Termination of treatment
			rmhTNF+cisplatin	Cisplatin alone		
Yuquan L 2005 [25]	18	18	rmhTNF 10 million units+NS 40 mL Cisplatin 60 mg + NS 50 mL	Cisplatin 60 mg + NS 50 mL	1/week	> 2 cycles, or pleural effusion disappeared
Do W 2005 [20]	31	31	rmhTNF 15 million units+NS 20 mL Cisplatin 30 mg/m^2^ + NS 20 mL	Cisplatin 40 mg/m^2^ + NS 20 mL	2–3/week	> 2 cycles, or pleural effusion disappeared
Yanhao W 2006 [13]	28	28	rmhTNF 15 million units+NS 40 mL Cisplatin 60 mg + NS 50 mL	Cisplatin 60 mg + NS 50 mL	1/week	> 2 cycles, or pleural effusion disappeared
Zhijun W [26]	53	31	rmhTNF 1.5 million units+NS 40 mL Cisplatin 60 mg + NS 50 mL	Cisplatin 60 mg + NS 50 mL	1/week	> 2 cycles, or pleural effusion disappeared
Shimeng F 2010 [22]	43	30	rmhTNF 15 million units+NS 20 mL Cisplatin 30 mg/m^2^ + NS 20 mL	Cisplatin 40 mg/m^2^ + NS 20 mL	2–3/week	> 2 cycles, or pleural effusion disappeared
Hua Z 2010 [9]	34	24	rmhTNF 1 million units+NS 20 mL Cisplatin 40 mg/m^2^ + NS 20 mL	Cisplatin 40 mg/m^2^ + NS 20 mL	1/week	> 2 cycles, or pleural effusion disappeared
Xiqiang W 2010 [24]	23	31	rmhTNF 2 million units+NS 40 mL Cisplatin 40 mg + NS 40 mL	Cisplatin 40 mg + NS 40 mL	1/week	> 2 cycles, or pleural effusion disappeared
Haiyan C 2015 [10]	26	27	rmhTNF 5 million units+NS 25 mL Cisplatin 40-60 mg + NS 50 mL	Cisplatin 40-60 mg + NS 25 mL	1/week	> 2 cycles, or pleural effusion disappeared
Jin L 2015 [12]	26	29	rmhTNF 15 × 10^6^ units+NS 20 mL Cisplatin 40 mg/m^2^ + NS 20 mL	Cisplatin 40 mg/m^2^ + NS 20 mL	1/week	> 4 cycles, or pleural effusion disappeared
Tao H 2016 [23]	30	30	rmhTNF 2 × 10^6^ units+NS 20 mL Cisplatin 30 mg/m^2^ + NS 20 mL	Cisplatin 30 mg/m^2^ + NS 20 mL	1/week	> 4 cycles, or pleural effusion disappeared
Chun W 2016 [19]	32	32	rmhTNF 3 million units+NS 60 mL Cisplatin 40 mg + NS 60 mL	Cisplatin 40 mg + NS 60 mL	1/week	> 2 cycles, or pleural effusion disappeared
Hua Z 2017 [21]	21	18	rmhTNF 1 million units+NS 20 mL Cisplatin 40 mg/m^2^ + NS 20 mL	Cisplatin 40 mg/m^2^ + NS 20 mL	1/week	> 4 cycles, or pleural effusion disappeared

*N* numbers of patients, *rmhTNF* recombinant mutant human tumor necrosis factor injection, *NS* normal saline

### Heterogeneity analysis of included studies does not show a statistical significance

The statistical value of heterogeneity was 5.06 (freedom =11) and I-squared is 0.0%, which indicated that there was no obvious heterogeneity among the studies. In addition, from a clinical design perspective, these studies had a good clinical homogeneity. So the fixed effects model of meta-analysis was used to finish the following analysis.

### Thoracic perfusion of rmhTNF combined with cisplatin shows a higher ORR and improves the QOL of MPE patients compared with cisplatin alone

**Fig. 2:**
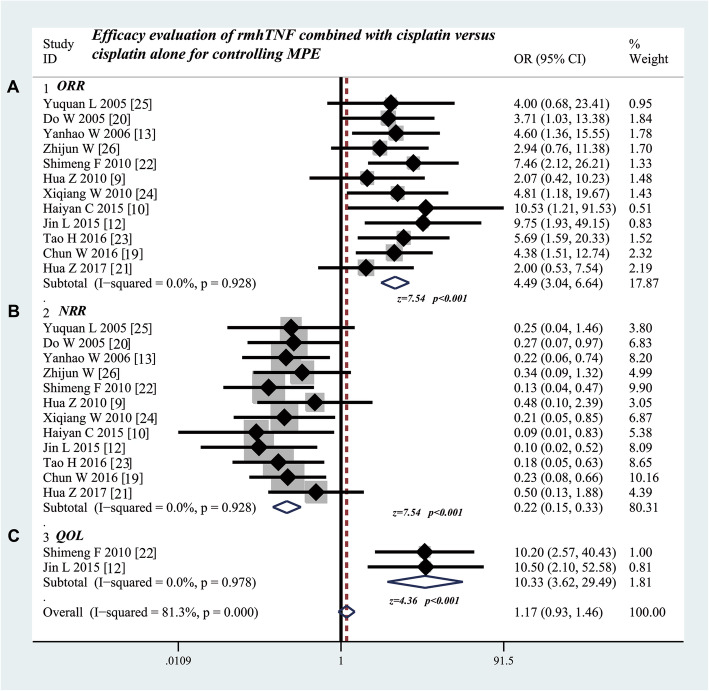
Efficacy evaluation of rmhTNF combined with cisplatin versus cisplatin alone through thoracic perfusion for treating MPE. **a** Thoracic perfusion of rmhTNF combined with cisplatin has a higher ORR compared with cisplatin alone. **b** Thoracic perfusion of rmhTNF combined with cisplatin has a lower ORR compared with cisplatin alone. **c** Thoracic perfusion of rmhTNF combined with cisplatin improves the QOL of patients with MPE compared with cisplatin alone. ORR, overall response rate; NRR, non-response rate; OR, odds ratio; QOL, quality of life; rmhTNF, recombinant mutant human tumor necrosis factor injection; MPE, malignant pleural effusion

As shown in Table 4, twelve studies [9, 10, 12, 13, 19–26] compared the ORR and NRR between the rmhTNF combined with cisplatin and cisplatin alone. The results showed that thoracic perfusion of rmhTNF combined with cisplatin had a higher ORR (odds ratio = 4.49; 95% CI 3.04 to 6.64; Z value = 7.54, P < 0.001) compared with cisplatin alone (Fig. 2a). The results on comparison of NRR also showed that thoracic perfusion of rmhTNF combined with cisplatin had a better efficacy (odds ratio = 0.22; 95% CI 0.15 to 0.33; Z value = 7.54, P < 0.001) than cisplatin alone (Fig. 2b). Two studies [12, 22] provided the data on comparing the QOL. The results showed that the odds ratio was 10.33 (95% CI 3.62 to 29.49, Z = 4.36, P < 0.001), suggesting that the presence of rmhTNF improved the QOL of patients with MPE (Fig. 2c).

**Table 4:** Efficacy evaluation of rmhTNF combined with cisplatin versus cisplatin alone through thoracic perfusion for treating MPE

Study	Study design (N)		Intravenous chemotherapy simultaneously	Pleural perfusion (N)		Efficacy of therapy								Improvement of SI (N,%)	
				Group 1	Group 2	Group 1				Group 2					
	Group 1	Group 2				CR	PR	SD	PD	CR	PR	SD	PD	Group 1	Group 2
Yuquan L 2005 [25]	18	18	No	rmhTNF+cisplatin	Cisplatin	5	11	3		2	10	6		–	–
Do W 2005 [20]	31	31	No	rmhTNF+cisplatin	Cisplatin	14	13	4		10	10	11		–	–
Yanhao W 2006 [13]	28	28	No	rmhTNF+cisplatin	Cisplatin	10	13	5		5	9	14		–	–
Zhijun W [26]	53	31	No	rmhTNF+cisplatin	Cisplatin	31	18	4		9	16	6		–	–
Shimeng F 2010 [22]	43	30	No	rmhTNF+cisplatin	Cisplatin	27	12	4		9	8	13		40/43	17/30
Hua Z 2010 [9]	34	24	TP	rmhTNF+cisplatin	Cisplatin	28	3	3		18	2	4		–	–
Xiqiang W 2010 [24]	23	31	No	rmhTNF+cisplatin	Cisplatin	2	18	3		1	17	13		–	–
Haiyan C 2015 [10]	26	27	No	rmhTNF+cisplatin	Cisplatin	6	19	1		4	15	8		–	–
Jin L 2015 [12]	26	29	No	rmhTNF+cisplatin	Cisplatin	16	8	1	1	7	9	8	5	24/26	16/29
Tao H 2016 [23]	30	30	No	rmhTNF+cisplatin	Cisplatin	14	12	4		8	8	14		–	–
Chun W 2016 [19]	32	32	No	rmhTNF+cisplatin	Cisplatin	16	8	8		7	6	19		–	–
Hua Z 2017 [21]	21	18	No	rmhTNF+cisplatin	Cisplatin	2	13	6		1	9	8		–	–

*N* cases, *rmhTNF* recombinant mutant human tumor necrosis factor injection, *Group 1* rmhTNF injection combined with cisplatin, *Group 2* cisplatin alone, *CR* complete response, *PR* partial response, *SD* stable disease, *PD* progressive disease, *TP* cisplatin in combination with paclitaxel

### Participation of rmhTNF increases the incidence rate of fever but does not affect the incidence of chest pain

**Fig. 3:**
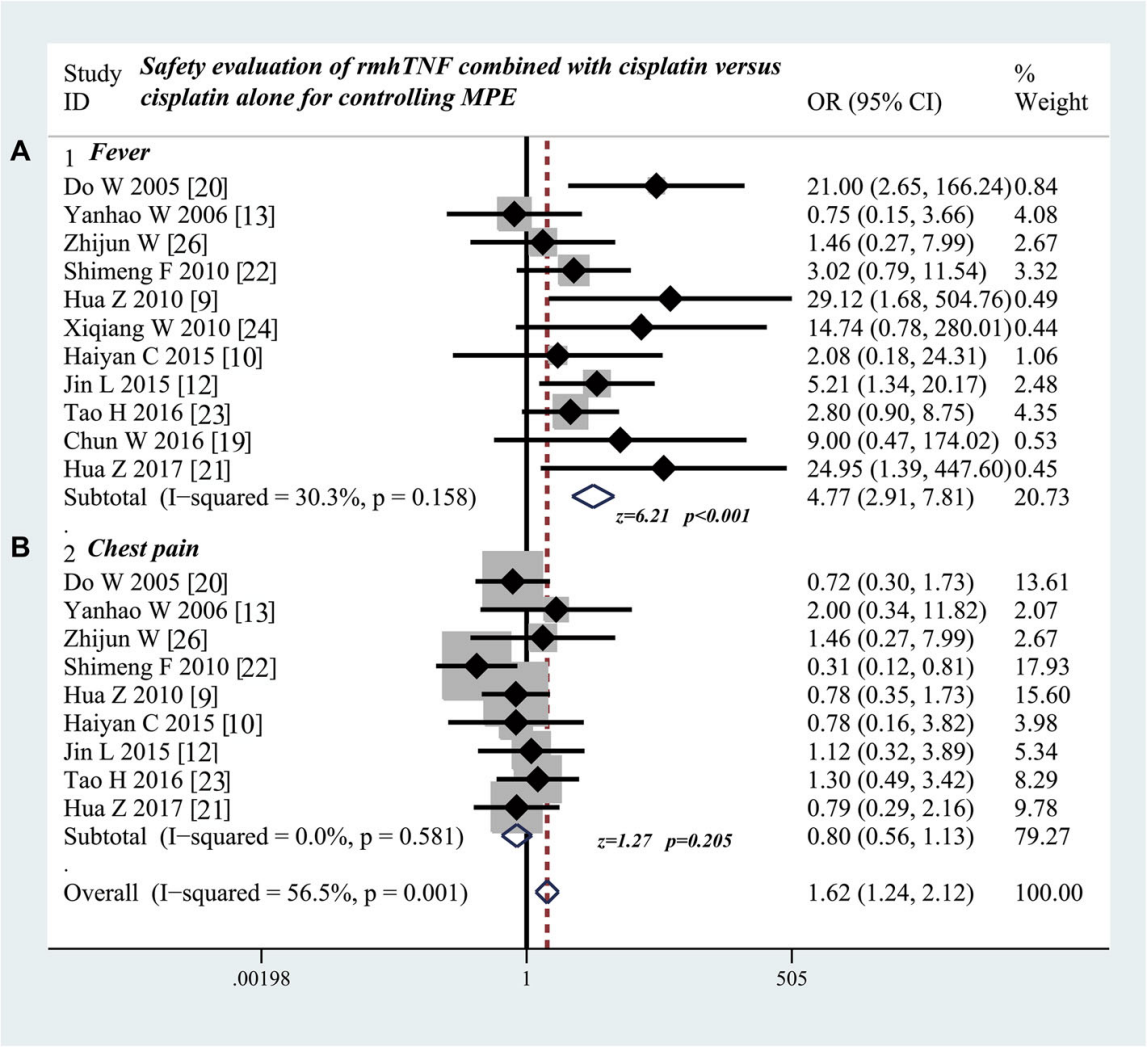
Safety evaluation of rmhTNF combined with cisplatin versus cisplatin alone through thoracic perfusion for treating MPE. **a** Combination of rmhTNF and cisplatin displays a higher incidence rate of fever than cisplatin alone. **b** Combination of rmhTNF and cisplatin has a similar incidence of chest pain with cisplatin alone. rmhTNF, recombinant mutant human tumor necrosis factor injection; MPE, malignant pleural effusion; OR, odds ratio

As shown in Table 5, eleven studies [9, 10, 12, 13, 19–24, 26] provided the data on comparing the incidence of fever. The results showed that the participation of rmhTNF increased the incidence rate of fever (odds ratio = 4.77; 95% CI 2.91 to 7.81; Z value = 6.21, P < 0.001) compared with cisplatin alone (Fig. 3a). Nine studies [9, 10, 12, 13, 20–23, 26] compared the incidence of chest pain. The odds ratio was only 0.80 (95% CI 0.56 to 1.13; Z value = 1.27, P = 0.205), indicating that the participation of rmhTNF did not increase the risk of chest pain (Fig. 3b).

**Table 5:** Safety evaluation of rmhTNF combined with cisplatin versus cisplatin alone through thoracic perfusion for treating MPE

Study	Fever (N)		Chest pain (N)		Myelosuppression (N)		Digestive reaction (N)		Liver and kidney dysfunction (N)	
	Group 1	Group 2	Group 1	Group 2	Group 1	Group 2	Group 1	Group 2	Group 1	Group 2
Do W 2005 [20]	21/30	1/30	13/30	18/30	11/30	10/30	17/30	14/30	1/30	1/30
Yanhao W 2006 [13]	3/28	4/28	4/28	2/28	0/28	2/28	0/28	16/28	–	–
Zhijun W [26]	5/53	2/31	5/53	2/31	5/53	3/31	4/53	4/31	2/53	2/31
Shimeng F 2010 [22]	13/43	3/30	8/43	18/30	2/43	5/30	3/43	15/30	–	–
Hua Z 2010 [9]	20/34	0/24	22/34	20/24	–	–	15/34	14/24	–	–
Xiqiang W 2010 [24]	5/23	0/31	–	–	8/23	9/31	16/23	21/31	2/23	2/31
Haiyan C 2015 [10]	2/26	1/27	3/26	4/27	2/26	3/27	4/26	2/27	–	–
Jin L 2015 [12]	14/26	3/29	6/26	6/29	1/26	1/29	4/26	4/29	2/26	1/29
Tao H 2016 [23]	14/30	5/30	13/30	10/30	–	–	7/30	9/30	–	–
Chun W 2016 [19]	4/32	0/32	–	–	–	–	–	–	–	–
Hua Z 2017 [21]	14/21	0/18	12/21	13/18	–	–	12/21	11/18	–	–
	P < 0.05		P > 0.05		P > 0.05		P > 0.05		P > 0.05	

*Group 1* rmhTNF injection combined with cisplatin, *Group 2* cisplatin alone

### Participation of rmhTNF does not increase the myelosuppression and hepatorenal toxicity but decreases the incidence of gastrointestinal adverse reaction

**Fig. 4:**
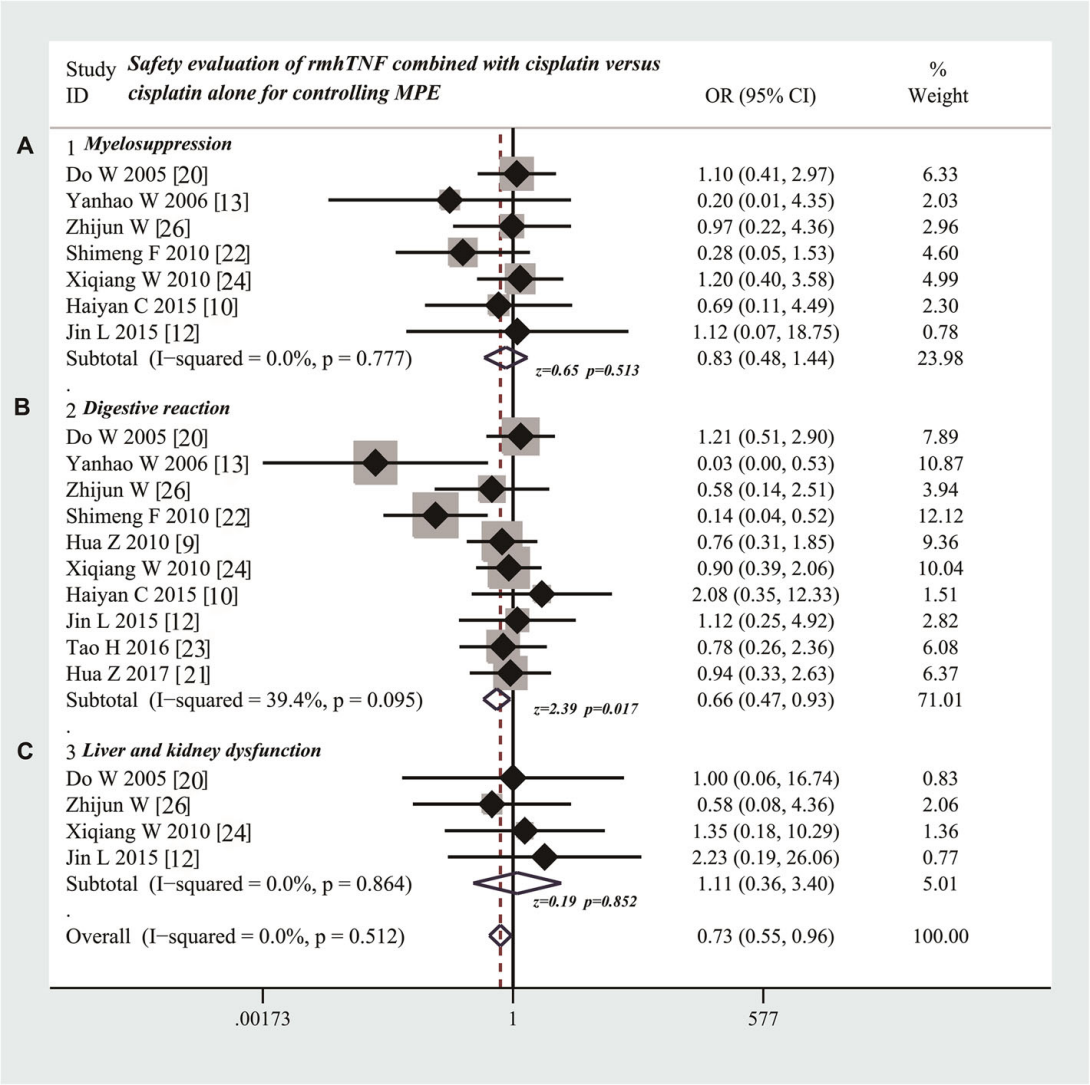
Safety evaluation of rmhTNF combined with cisplatin versus cisplatin alone through thoracic perfusion for treating MPE. **a** Combination of rmhTNF and cisplatin does not increase incidence of myelosuppression compared with cisplatin alone. **b** Combination of rmhTNF and cisplatin decreases the incidence of gastrointestinal side reaction compared with cisplatin alone. **c** Combination of rmhTNF and cisplatin does not increase the incidence of liver and kidney dysfunction compared with cisplatin alone. rmhTNF, recombinant mutant human tumor necrosis factor injection; MPE, malignant pleural effusion; OR, odds ratio

As shown in Table 5, seven [10, 12, 13, 20, 22, 24, 26] studies compared the incidence rate of myelosuppression. The results showed that the participation of rmhTNF did not increase the incidence rate of myelosuppression (odds ratio = 0.83; 95% CI 0.48 to 1.44; Z value = 0.65, P = 0.513) compared with cisplatin alone (Fig. 4a). Ten studies [9, 10, 12, 13, 20–24, 26] compared the incidence rate of gastrointestinal adverse reaction. The results suggested that the presence of rmhTNF decreased the incidence of gastrointestinal adverse reaction (odds ratio = 0.66; 95% CI 0.47 to 0.93; Z value = 2.39, P = 0.017) compared with cisplatin alone (Fig. 4b). Four studies [12, 20, 24, 26] compared the incidence rate of liver and kidney dysfunction. The results showed that the adding of rmhTNF did not increase the incidence of liver and kidney dysfunction (OR = 1.11; 95% CI 0.36 to 3.40; Z value = 0.19, P = 0.852) compared with cisplatin alone (Fig. 4c).

### Sensitivity and publication bias analysis of included trials

**Fig. 5:**
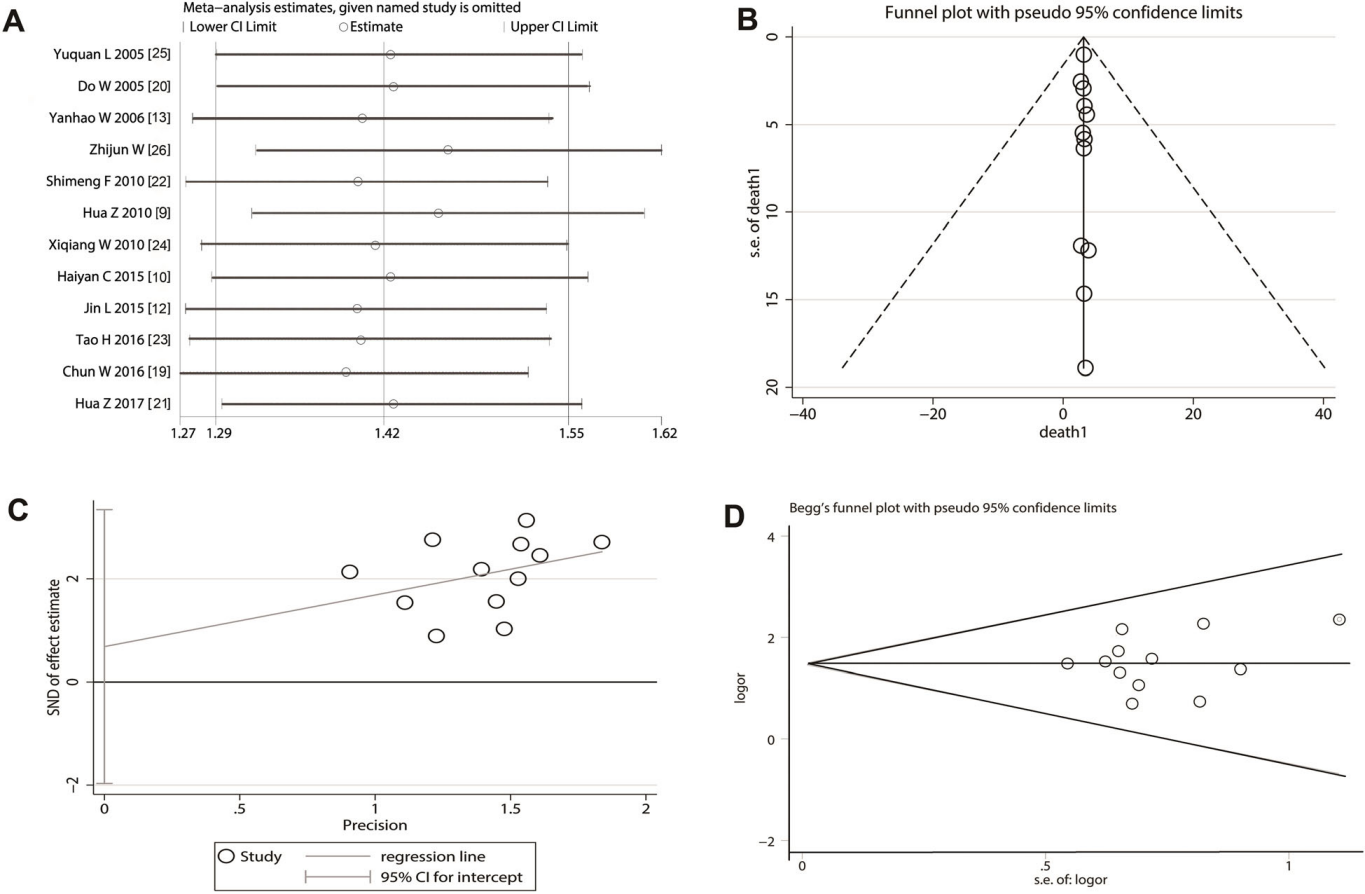
Sensitivity analysis and publication bias evaluation on included studies. **a** The deletion of any study does not shake the overall effect of meta-analysis. **b** The vertical funnel plot of meta-analysis shows that each study is located on the central axis. **c** The distribution graph of Egger’s test shows that included studies are precisely distributed on both sides of the baseline. **d** The funnel plot derived from the Begg’s test is nearly symmetrical

Sensitivity analysis of included studies showed that the deletion of any study did not shake the overall effect of meta-analysis and the mixed estimate for the included study was 1.4172184 (95% CI = 1.29167 to 1.554978) (Fig. 5a). The vertical funnel plot of included studies showed that all studies were located on the central axis (Fig. 5b). The distribution graph derived from Egger’s test showed that included studies were precisely distributed on both sides of the baseline (Fig. 5c) (P > |t| = 0.579; 95% CI: − 1.970162 to 3.33541; T = 0.57). The Std. Dev. of Score from the Begg’s test was 14.58 (Pr > |z| = 0.95) and the horizontal funnel plot derived from the Begg’s test was symmetrical (Fig. 5d). These results indicated that included trials did not show a potential publication bias.

## Discussion

Because most patients with lung cancer often suffer from MPE, which causes a decrease in QOL and even shortens life expectancy, the treatment of MPE is a thorny issue for clinicians [5]. Studies have shown that TNF-alpha can inhibit the production and accumulation of MPE and prolong the survival of fibrosarcoma-bearing mice [21, 27–29]. Although systemic application of rmhTNF has a certain anti-tumor effect on patients with advanced cancer, it causes a vomiting and hypotension, so current research has still focused on the local treatment [29, 30]. In China, application of rmhTNF for the treatment of MPE by way of thoracic perfusion has been suggested as an effective method. We performed a systemic review and meta-analysis to quantify the efficacy and safety of rmhTNF in the treatment of MPE caused by lung cancer. A total of 12 studies [9, 10, 12, 13, 19–26] were included in this meta-analysis. We found that the included studies had a good comparability and homogeneity, so we used the fixed effects model of meta-analysis to carry out the analysis of all data.

Our study showed that thoracic perfusion of rmhTNF combined with cisplatin had a higher ORR compared with cisplatin alone, suggesting that presence of rmhTNF significantly increased the therapeutic effect of MPE. The biological activity of 70% ~ 95% of the total TNF activity exerted by TNF-α [27, 30] and previous studies have suggested that TNF-α can be used to treat soft tissue sarcomas and metastatic melanoma [28, 31]. In recent years, TNF-α modified by asparagine glycine arginine (NGR) is used to treat colorectal cancer, liver cancer and malignant pleural mesothelioma [32]. Compared to natural tumor necrosis factor, the rmhTNF produced in China knocks out the first seven amino acid residues of the N-terminus of TNF-α and simultaneously replaces amino acids 8, 9, 10, and 157. Experimental study shows that the anti-tumor activity of modified rmhTNF was 10 times higher than that of the wild type and the toxicity was reduced by 5 times [33]. Nowadays, health-related quality of life (QOL) has become the main indicator of tumor clinical treatment. Under the premise of the same curative effect (if the survival time is the same), the treatment method that can improve QOL is more recommended [4]. Our study showed that the combination perfusion of rmhTNF plus cisplatin improved the QOL of patients with MPE compared with cisplatin alone, indicating that the rmhTNF has played a role in improving QOL.

We found that the most common AEs in two different treatment options were fever, chest pain, myelosuppression, digestive reaction and liver and kidney dysfunction. We specially found that presence of rmhTNF seemed to increase the incidence rate of fever but did not increase the incidence of chest pain, myelosuppression and liver and kidney dysfunction. However, participation of rmhTNF obviously decreased the incidence of digestive adverse reaction. Previous study points out that the AEs of rmhTNF used for local thoracic perfusion is similar to that of similar drugs, but compared with the reported intravenous administration, the incidence of AEs in the thoracic perfusion is significantly decreased [33]. As an endogenous heat source, TNF may cause fever by directly stimulating hypothalamus temperature regulation center and stimulating macrophages to release IL-1 and IL-6 [34, 35]. It is reported that the TNF stimulates the metabolism of arachidonic acid in cells and increases the synthesis of prostaglandins, prostacyclins, and hemoglobin A2 in the cyclooxygenase metabolic pathway, it can pass the blood brain barrier to the temperature regulating center of hypothalamus, and release the arachidonic acid, thus induces the synthesis of prostaglandins and cause fever [35, 36]. Study has shown that pretreatment with dexamethasone before treatment of the rmhTNF can significantly reduce the incidence of fever [33].

Sensitivity analysis can not only evaluate the stability and reliability of the combined results of meta-analysis, but also assess whether the combined results is affected by a single study. In our study, sensitivity analysis showed that the deletion of any study did not shake the overall effect of meta-analysis. Because positive results are more likely to be published, some articles with negative results may not be published, which will lead to overestimate and underestimate the true statistical effects. In our study, we found that the vertical funnel plot of meta-analysis, Egger’s test and Begg’s test all indicated that included studies did not have a potential publication bias.

However, we also found some flaws in included studies. First, most of included studies did not perform the blind and allocation concealment, which may affect the level of evidence. Second, the size of some investigations is relatively small, which may weak the efficacy of statistics. Third, included studies did not perform a subgroup analysis of the efficacy of rmhTNF on different histological types of lung cancer. Future research should focus on this topic. Even so, these studies still provided a significant conclusion that the rmhTNF may be a good adjunct in the treatment of MPE. Of course, rmhTNF, as a new drug, has still many unknown problems that need to be answered through further investigations. Before rmhTNF is extensively recommended for use in clinic, large sample and multicenter double-blind randomized controlled trials are still needed to confirm this conclusion.

## Conclusion

Thoracic perfusion of rmhTNF combined with cisplatin has a better ORR in the treatment of MPE and improves the QOL of MPE patients, as compared with cisplatin alone. Although the participation of rmhTNF increased the incidence of fever, it reduced the adverse reactions in the digestive tract.

## Data Availability

The datasets supporting the conclusions of this article are included within the article.

## Abbreviations

ADSC: Adenosquamous carcinoma
AEs: Adverse effects
cDNA: Complementary DNA
CNKI: China National Knowledge Infrastructure
CR: Complete response
DCR: Disease control rate
IL-1: Interleukin-1
IL-6: Interleukin-6
LAC: Lung adenocarcinoma
LSCC: Lung squamous cell carcinoma
MPE: Malignant pleural effusion
NGR: Asparagine glycine arginine
NRR: No response rate
OR: Odds ratios
ORR: Objective response rate
PD: Progressive disease
PR: Partial response
PRISMA: Preferred Reporting Items for Systematic Reviews and Meta-Analyses
QOL: Quality of life
rmhTNF: Recombinant mutant human tumor necrosis factor
SCLC: Small cell lung cancer
SD: Stable disease
SFDA: China State Food and Drug Administration
SI: Symptom improvement
TNF: Tumor necrosis factor
